# A Salivary Odorant-Binding Protein Mediates *Nilaparvata lugens* Feeding and Host Plant Phytohormone Suppression

**DOI:** 10.3390/ijms22094988

**Published:** 2021-05-08

**Authors:** Hao Liu, Chao Wang, Chang-Lai Qiu, Jin-Hua Shi, Ze Sun, Xin-Jun Hu, Le Liu, Man-Qun Wang

**Affiliations:** Hubei Insect Resources Utilization and Sustainable Pest Management Key Laboratory, College of Plant Science and Technology, Huazhong Agricultural University, Wuhan 430070, China; haoliu2020@webmail.hzau.edu.cn (H.L.); chaowang@webmail.hzau.edu.cn (C.W.); qiucl@webmail.hzau.edu.cn (C.-L.Q.); shijinhua@webmail.hzau.edu.cn (J.-H.S.); szzy.0212@163.com (Z.S.); xinjunh@webmail.hzau.edu.cn (X.-J.H.); liule@webmail.hzau.edu.cn (L.L.)

**Keywords:** host interaction, *Nilaparvata lugens*, odorant-binding proteins, plant defense, salivary gland protein

## Abstract

Odorant-binding proteins (OBPs) typically act as transporters of odor molecules and play an important role in insect host location. Here, we identified an OBP in brown planthopper (BPH) *Nilaparvata lugens* salivary glands via transcriptome sequencing. Real-time quantitative PCR and Western blotting analysis results showed that NlugOBP11 was highly expressed in salivary glands and secreted into rice plant during feeding, suggesting that it assists in BPH feeding on rice. Functional analysis in *N. lugens* saliva revealed that silencing this gene by RNA interference decreased the BPH stylet performance in the phloem of rice plants, reduced sap sucking, and ultimately led to insect death. Moreover, overexpression of NlugOBP11 in rice protoplasts or *Nicotiana benthamiana* leaves inhibited the production of defense-related signaling molecule salicylic acid in rice plant. The results demonstrate that NlugOBP11 is not only essential for BPH feeding, but also acts as an effector that inhibits plant defense.

## 1. Introduction

When herbivorous insects feed on plants, their saliva and oral secretions can be identified by the host plants [1,2]. Plant immune signaling pathways become activated to protect the host plant [3,4,5]. For example, plants can develop calluses to prevent insects from feeding on phloem [6]. Regarding chewing insects, plants can recognize fatty acid-amino acid conjugates (FACs) present in herbivore saliva, as well as express defense-related genes and produce defensive chemicals [7,8,9]. In response to *Pieris brassicae* larvae, a β-glucosidase induces plants to release volatile organic compounds that attract its predators [10]. Similarly, in *Nicotiana attenuata*, oral secretions from *Manduca sexta* activate plant mitogen-activated protein kinase (MAPK), as well as the jasmonic acid (JA) and ethylene pathways [4,7]. Many plants produce salicylic acid (SA) and JA signaling molecules that stimulate plant defenses against herbivores [11,12,13].

Insect saliva contains elicitors that induce plant resistance and effectors that inhibit plant defenses. For example, the C002 protein (expressed in saliva, and secreted into host plants during feeding) is indispensable for feeding by the pea aphid (*Acyrthosiphon pisum*) [14], and overexpression of C002 in *Nicotiana benthamiana* increases the fecundity of green peach aphid (*Myzus persicae*) [15]. Numerous effectors have been identified in saliva. In vetch aphid (*Megoura viciae*), the saliva contains calcium-binding proteins that induce dispersed forisomes to revert back to the nonplugging contracted state and promote aphid feeding [16]. In *Myzus persicae*, the MP10 protein suppresses oxidative bursts [15]. Similarly, MpMIF (a migration inhibitory factor (MIF) in *Myzus percicae*) in aphid watery saliva is secreted into plants, where it can affect JA and SA signaling pathways [17]. In recent years, several saliva proteins have been studied in brown planthopper (BPH, *Nilaparvata lugens*), a devastating rice pest that causes serious economic losses throughout Asia [18,19]. The BPH sheath protein *N. lugens*-secreted mucin-like protein (NLMLP) is required for BPH feeding and can induce plant cell death and transcription of pathogen-responsive genes [20]. Ji et al. [21] found that Endo-b-1,4-glucanase (an endoglucanase present in BPH watery saliva that is secreted into host plants during feeding) is an effector that helps BPH to circumvent plant defenses. There are numerous proteins in insect saliva, but their functions remain poorly understood.

Recently, salivary gland transcriptome sequencing of BPH has identified numerous odorant-binding proteins (OBPs) [22]. OBPs are ~16 kDa soluble proteins with six conserved cysteine residues that form three disulphide bonds, and a tertiary structure comprising six α-helices forming a stable hydrophobic cavity [23,24,25,26]. This capsule-like structure binds hydrophobic odor molecules in the lymph and transports them to odor receptors, resulting in changes to insect behavior [24,27]. Previous research on OBPs has mainly focused on their functions in the antennas, where they play an important role in insect mating and locating partners, as well as host location and avoiding natural enemies [28,29,30].

Our laboratory has conducted numerous studies on the structure of OBPs, the binding and release mechanisms of odorant molecules, and host localization in insects [31,32,33]. We found that OBPs are not only present in the antennae, but also in other tissues without olfactory functions. D7-related OBPs are present in hematophagous insect saliva that bind cysteinyl-leukotrienes and biogenic amines to reduce inflammation [34,35,36]. Blowfly (*Phormia regina*) saliva contains PregOBP56a, which solubilizes fatty acids during feeding and helps deliver the fatty acids to the midgut [37].

As a typical sucking insect, the BPH feeding patterns have been well studied by an electrical penetration graph (EPG) [38]. Based on the location of the stylets and honeydew excretion, the EPG waveforms for the stylet penetration behaviors of BPH were classified into five types, representing different types of insect feeding behavior: non-penetration of stylets (np, the stylet was outside the plant tissue, BPH insects resting on plants, walking or searching for feeding sites), pathway (ph, BPH insects used their stylets to search for the target cells in plant tissues in a series of activities, including penetrating plant cells, salivating, tasting, and forming branches of the stylet sheath), an intracellular activity in phloem region (N4-a, the stylet penetrated the vascular bundle of the rice plant and secrete watery saliva), phloem sap ingestion (N4-b, BPH obtain the phloem nutrients), xylem ingestion (N5, BPH obtain the xylem nutrients) [6,38,39].

Notably, our laboratory also discovered OBPs in BPH salivary glands via transcriptome sequencing, and their functions in insect saliva are intriguing. In the present study, we identified a new OBP (NlugOBP11) in BPH salivary glands. This gene is highly expressed in salivary glands and is indispensable for insect survival. The difficulty of silencing BPH in feeding on rice was verified by EPG. The results revealed that NlugOBP11 not only assists BPH feeding, but also inhibits SA signaling in host plants.

## 2. Results

### 2.1. NlugOBP11 Sequences, Expression Patterns and Localization

**Figure 1:**
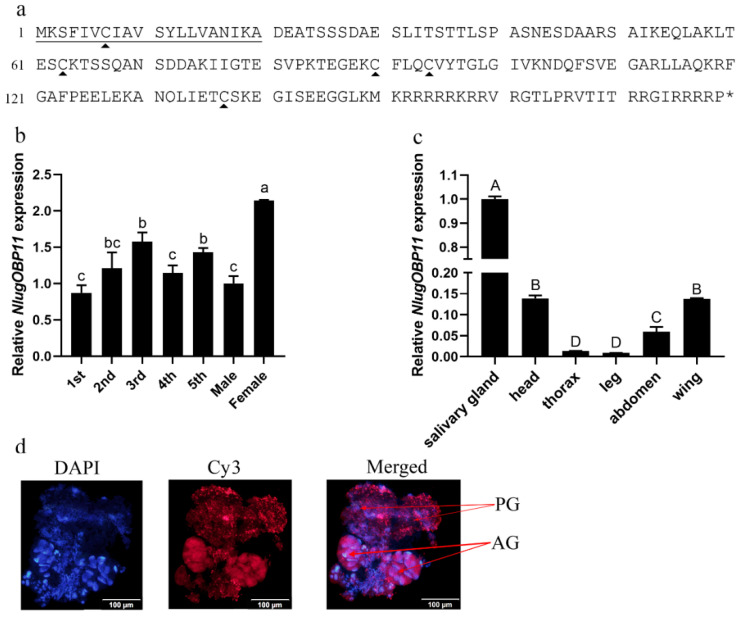
Molecular characterization of NlugOBP11. (**a**) Amino acid sequence of OBP11. The solid underline indicates the signal peptide. The asterisk indicates the stop codon. The trigonometric symbols indicate cysteine. (**b**) Expression patterns of *NlugOBP11* at different developmental stages (1st to 5th, first to fifth instar; F, female adult; M, male adult). (**c**) Expression patterns of *NlugOBP11* in different tissues (salivary gland, head without salivary glands, thorax, abdomen, legs, and wings were dissected from BPH adults), *β-actin* was used as reference control. The data are mean ± SEM, three repeats; different letters above the bars indicate significant differences, as determined by one-way ANOVA significant difference test (*p* < 0.05). The differences between the means were determined by Tukey’s HSD test. (**d**) Immunohistochemical (IHC) staining of NlugOBP11 in the BPH salivary gland. Nucleus (DAPI, blue) and NlugOBP11 (Cy3-labeled secondary antibody, red) staining is displayed. PG, principal glands; AG, accessory glands; bar = 100 µm.

We obtained a full-length cDNA of the NlugOBP11 gene by PCR based on the BPH salivary gland transcriptome. The cDNA contained an ORF of 540 bp encoding a polypeptide of 179 amino acid residues (Figure 1a). The protein possessed a secretory signal peptide (first 20 amino acids) and has no transmembrane domains, indicating potential secretion. Amino acid sequence alignment followed by phylogenetic tree analysis revealed that NlugOBP11 is homologous to other insect OBPs (Supplementary Figures S1 and S2) NlugOBP11 shared the highest homology (100%) with OBP from delphacidae *(Laodelphax striatellus*, AGZ04923; *Sogatella furcifera*, AHB59655) followed by that from Aphididae (*Myzus persicae*; ACI30682 (66%)) and Acrididae (*Locusta migratoria*; ACI30696 (58%)). To explore the functions of NlugOBP11, mRNA levels were analyzed in different tissues of BPH at different developmental stages by qPCR. The results showed that NlugOBP11 was expressed in all stages, and more highly expressed in salivary glands than in other tissues (Figure 1b,c). The distribution of NlugOBP11 in salivary glands was analyzed by immunohistochemical (IHC) staining, and IHC signals were detected in the principal glands and salivary ducts, but not in the accessory glands (Figure 1d). These results suggest that NlugOBP11 probably play a key role in feeding.

### 2.2. NlugOBP11 Is Secreted into Rice during BPH Feeding

**Figure 2:**
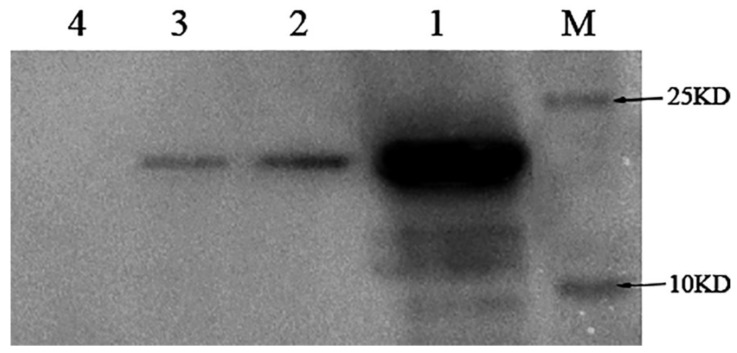
Detection of the protein NlugOBP11 in rice extract after nymphs feeding by Western blots. Protein samples for Western blot analysis were as follows. M: Mark; lan1: NlugOBP11 protein (positive control); lan2: extracts from rice plants after nymph feeding; lan3: extract from the salivary glands; lan4: extracts from rice plants without nymph feeding.

In order to investigate whether NlugOBP11 is secreted into rice plants during feeding, 300 fifth instar nymphs were placed on rice stems for 24 h and then removed. Outer leaf sheath proteins were extracted and Western blotting analysis was performed using polyclonal anti-NlugOBP11 rabbit antibodies. The results revealed a band of ~25 kDa for plants infested with BPH (Figure 2, line 2), BPH salivary glands (Figure 2 line 3) and positive controls (Figure 2, line 1), but not for rice plants without nymphs (Figure 2, line 4). These results indicated the NlugOBP11 protein was secreted from salivary glands into rice plants during BPH feeding.

### 2.3. NlugOBP11 Is Indispensable for BPH Growth

**Figure 3:**
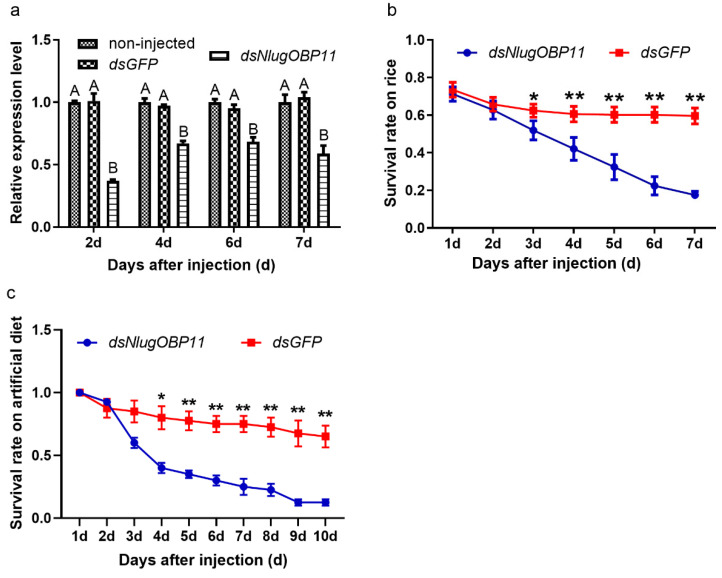
Knocking down NlugOBP11 decreases survival rates among BPH nymphs. (**a**) The *NlugOBP11* transcript levels after injecting dsRNA in whole bodies on different days. The data are mean ± SEM (*n* = 3). Different letters above the bars indicate significant differences between the means as determined by Tukey’s HSD test (*p* < 0.01, one-way ANOVA test). (**b**) The survival rates of nymphs after injected dsRNA feeding on rice. (**c**) The survival rates of nymphs after injected dsRNA feeding on rice or artificial diet. The data are mean ± SEM (*n* = 4). Asterisks show significant differences from the control group (* *p* < 0.05; ** *p* < 0.01; Student’s t test).

In order to confirm that *NlugOBP11* was successfully silenced, expression of *NlugOBP11* was analyzed by qPCR after injection. The results showed that *NlugOBP11* gene expression was decreased by more than 40% compared with controls (Figure 3a). To investigate the effect of NlugOBP11 on the growth and development of BPH, insects were injected with dsRNA, and then reared on TN1 rice seedlings or artificial diet, and the survival rate was measured. After 3 days, the BPH survival rate was significantly lower than that of the control group; it was <25% at 7 days post-injection for both rice seedling and artificial diet groups (Figure 3b,c). There were no differences in survival rate of BPH between those reared on rice seedling and artificial diet groups. These results demonstrate that NlugOBP11 is indispensable for BPH survival.

### 2.4. NlugOBP11 Affects BPH Feeding Behavior

**Figure 4:**
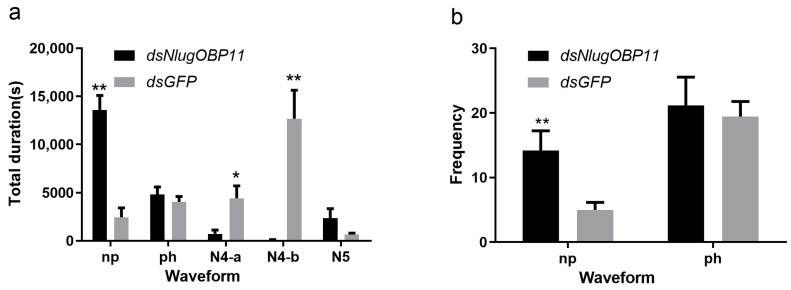
Knocking down NlugOBP11 reduces the BPH nymphs feeding. Feeding behavior was recorded for 6 h by electrical penetration graph (EPG). (**a**) Total duration of waveform; (**b**) frequency of waveform. Waveforms: np (non-penetration), ph (pathway waveform), N4-a (sieve element salivation), N4-b (ingestion of phloem). The data are mean ± SEM (*n* = 10). Asterisks show significant differences from the control group (* *p* < 0.05; ** *p* < 0.01; Student’s t test).

Following the gene silencing and survival experiments, at 2 days after injection, nymphs were selected for profiling of feeding and foraging using the EPG technique. EPG analysis showed that insects lacking NlugOBP11 could not obtain the phloem nutrients normally (Figure 4). The frequency and total duration of the np waveforms were significantly increased, compared with those of the control group (Figure 4a,b, np), but the total duration of the secretion of saliva and ingestion in the phloem were significantly reduced compared with controls (Figure 4a, N4-a and N4-b). The total duration of the ph and N5 waveforms were not statistically significant, compared with those of the control group (Figure 4a,b, ph and N5). These results showed that NlugOBP11 only affect stylet performance in phloem and required by BPH in obtaining the phloem nutrients.

### 2.5. Secretion of NlugOBP11 Suppresses the Defense Responses of Rice

**Figure 5:**
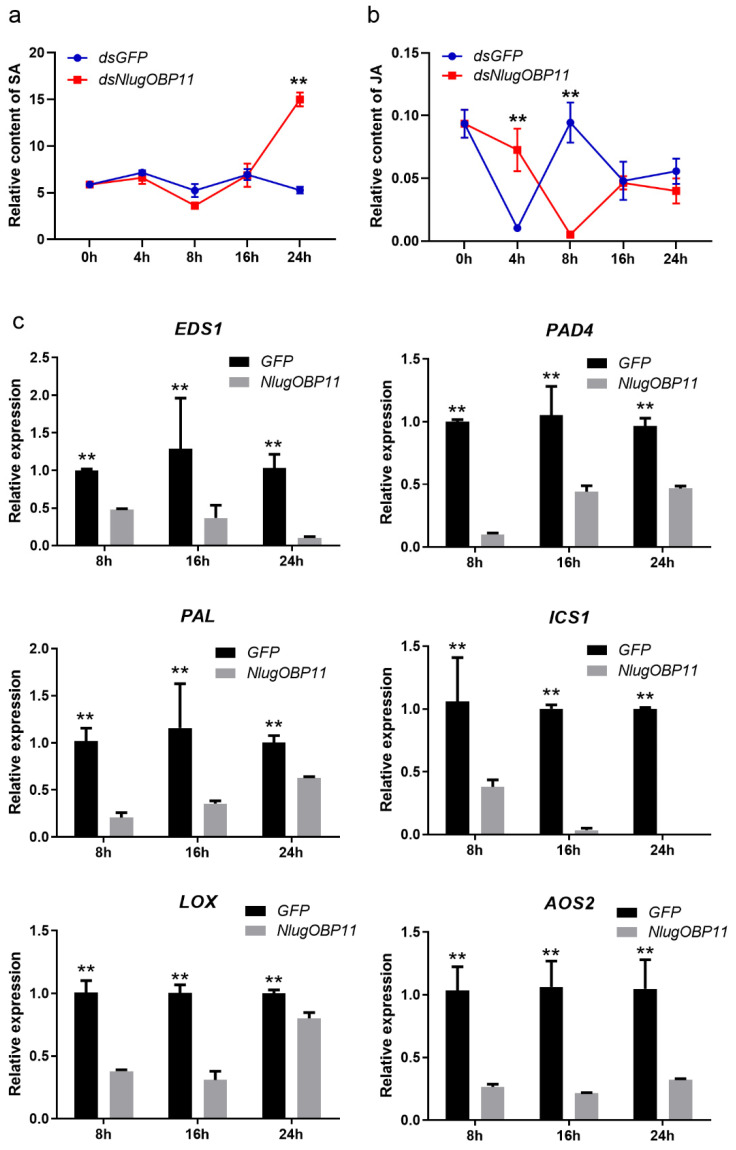
NlugOBP11 secreted by BPH decreased the levels of salicylic acid (SA) and jasmonic acid (JA) in rice. (**a**) The relative content of SA in dsRNA-injected and nymph-damaged rice plants. (**b**) The relative content of JA in dsRNA-injected and nymph-damaged rice plants. The relative content of phytohormones was obtained through dividing the peak area of the phytohormones by the corresponding internal standard peak area. (Data are mean ± SEM, *n* = 4–9.) (**c**) The SA and JA relative gene transcript levels when overexpressing NlugOBP11 in rice protoplasts. *EDS1, PAD4, PAL*, and *ICS1* are the SA synthesis-related genes. *LOX* and *AOS2* are the JA synthesis-related genes. Rice *GAPDH* was used as reference control. Asterisks show significant differences from the control group (data are mean ± SEM, *n* = 3; ** *p* < 0.01; Student’s t test).

SA and JA are important phytohormones in rice defenses against insect herbivores [40,41]. To determine whether the salivary protein NlugOBP11 affects the production of these hormones, we investigated the levels of JA and SA in rice plant after BPH nymph feeding, and NlugOBP11 silenced. The results showed that SA levels increased at 24 h in NlugOBP11-silenced nymph plants (Figure 5a). By contrast, compared with controls, JA levels were increased at 4 h but decreased at 8 h in NlugOBP11-silenced nymph plants (Figure 5b).

Transcription level genes related to JA and SA synthesis in rice protoplast wherein NlugOBP11 was overexpressed were also examined. Four SA- and two JA-related genes were investigated by qPCR. When overexpressing NlugOBP11 in protoplasts, SA synthesis-related genes *EDS1* (enhanced disease susceptibility 1), *PAD4* (phytoalexin deficient 4), *PAL* (phenylalanine ammonia-lyase) and *ICS1* (isochorismate synthase 1) [42] were downregulated compared with the control group (Figure 5c). This was also the case for JA synthesis-related genes *LOX* (lipoxygenase) and *AOS2* (allene oxide synthase 2) [42,43].

**Figure 6:**
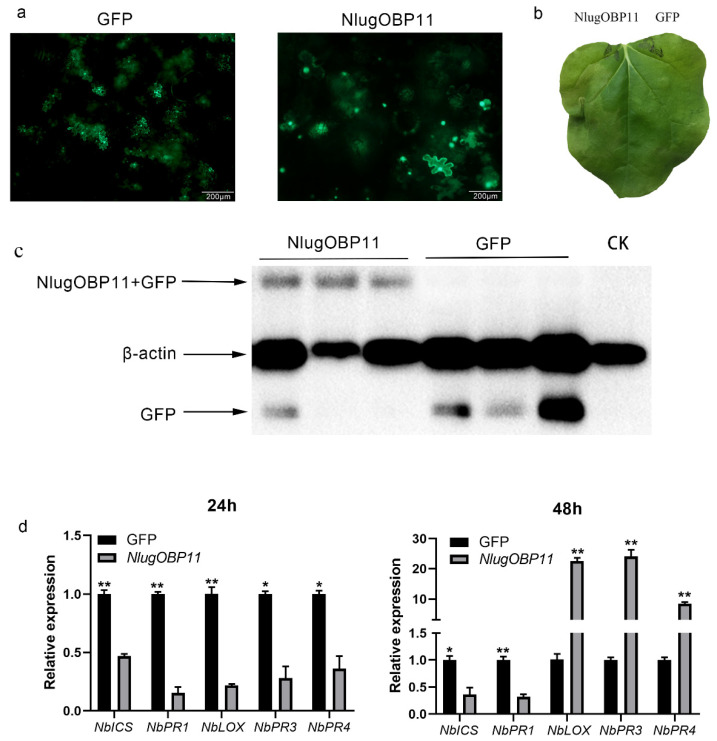
NlugOBP11 affects plant defense responses during transfection. (**a**) Images of *N. benthamiana* leaf cells morphology 2 d after transformation with GFP or NlugOBP11. (**b**) Leaves of *N. benthamiana* were photographed 4 d after agroinfiltration. (**c**) Western blot analysis of proteins from *N. benthamiana* leaves transformed with GFP and NlugOBP11 at 2 days. Line CK: extract proteins from normal *N. benthamiana* leaves; line NlugOBP11: extract proteins from *N. benthamiana* leaves transiently expressing NlugOBP11 with GFP tag; line GFP: extract proteins from *N. benthamiana* leaves transiently expressing GFP. (**d**) SA and JA relative gene transcript levels after overexpressing NlugOBP11 in *N. benthamiana* leaves. *NbICS* and *NbPR1* are the SA synthesis-related genes. *NbLOX, NbPR3* and *NbPR4* are the JA synthesis-related genes. *N. benthamiana L25* was used as the reference control. Asterisks show significant differences from the control group (data are mean ± SEM, *n* = 3; * *p* < 0.05; ** *p* < 0.01; Student’s t test).

We also verified that NlugOBP11 suppressed the defense responses in *N. benthamiana* plants. The *N. benthamiana* leaf cells were abnormal on the second day (Figure 6a), and watery lesion on leaves was obvious on the fourth day (Figure 6b) when overexpressing NlugOBP11 protein (Figure 6c). In other studies, pathogen resistance (PR) gene *NbPR1* typically activates the SA signaling pathway, while *NbPR3* and *NbPR4* genes are associated with the JA-dependent response [17,44]. Overexpressing NlugOBP11 in *N. benthamiana* leaves downregulated the SA synthesis-related genes *NbICS* and *NbPR1* at 24 and 48 h and the JA-related genes *NbLOX*, *NbPR3* and *NbPR4* at 24 h but increased at 48 h when compared with the control group (Figure 6d).

## 3. Discussion

Odorant-binding proteins are small proteins widely distributed in insects that help them to find host plants and mates. Recent research has revealed novel functions for OBPs in mating and development [45,46], in addition to their olfactory functions. OBPs are present in the salivary glands of insects [22,47], but their functions remain unclear.

In the present work, we identified an OPB in BPH with potentially novel functions. NlugOBP11 was found to be abundant in the salivary glands and secreted into rice during feeding. Although, *NlugOBP11* was expressed in the tissues of all developmental stages, expression level was highest in salivary glands (more than 10-fold higher). Silencing of NlugOBP11 ultimately resulted in insect death, indicating its indispensable function for the growth and development of BPH. Our results suggest that the major functions of NlugOBP11 are related to feeding and probably interact with rice.

EPG is a powerful method for analyzing the probing and feeding behaviors of sucking insects [6,48]. There are five typical waveforms; non-penetration (np), pathway (ph), sieve element salivation (N4-a), ingestion in phloem (N4-b) and xylem sap ingestion (N5) [6,38]. Through EPG tests, we found that NlugOBP11 decreased the BPH stylet performance in rice plants. The total duration of the N5 and ph waveforms was the same as the control group. Thus, silencing NlugOBP11 did not impact the function of BPH stylet probing and sucking. By contrast, the duration of N4-a and N4-b waveforms was significantly lowered than those of the control group, and only few nymphs could suck phloem normally. Furthermore, the stylets of NlugOBP11-silenced nymphs remained still for a long time (np waveform). These findings demonstrate that NlugOBP11 only affected stylet performance in phloem.

Plants produce phytohormones to help resist insect feeding. In this experiment, we confirmed that the NlugOBP11 was secreted into rice during BPH feeding. We hypothesized that the NlugOBP11 assists nymph feeding by altering plant phytohormones (SA and JA) content. It was shown that NlugOBP11-silenced nymph-fed plants elicited an increase in SA content at 24 h and increased JA at 4 h but decreased at 8 h. Furthermore, overexpression of NlugOBP11 in rice protoplasts or *N. benthamiana* leaves suppressed the expression of SA synthesis-related genes. However, JA-related genes were only inhibited at 24 h but increased at 48 h. The upregulation of JA-related genes coincided with downregulation of SA-related genes in *N. benthamiana*. Numerous studies revealed that SA and JA have antagonistic effects especially in *N. benthamiana* and *Arabidopsis thaliana* plants [49,50,51], and these antagonistic effects are also related to the interaction time and concentration [52,53,54]. The amount of NlugOBP11 secreted into plants during BPH feeding was lower than the amount overexpressed in *N. benthamiana.* Therefore, NlugOBP11 might not trigger elevated JA content during the BPH feeding, during which time a decrease in SA content is beneficial to the performance of piercing sucking insects [55]. These results suggest that NlugOBP11 helps BPH to inhibit the plant defense system by suppressing the SA content.

BPH was also unable to survive in rice and article diet with silenced NlugOBP11. NlugOBP11 is indispensable during BPH feeding, and its possible inhibition of SA synthesis suggests an additional function of this gene. Unlike the BPH which cannot form salivary sheath and cannot reach the phloem, the BPH without NlugOBP11 is able to reach the phloem but is devoid of feeding [56]. Since OBP is a protein that transports odor molecules, we wonder whether it plays a role in transporting nutrients during the feeding process. Nevertheless, phylogenetic tree analysis indicated that this protein has high homology with that of the Delphacidae and Aphididae. Whether it is ubiquitous in piercing and sucking insects and plays pivotal role in the feeding process is an aspect for further studies.

In summary, our study showed that NlugOBP11 is an indispensable protein for BPH feeding, which also reduces the transcription levels of genes related to SA synthesis in rice, and consequently lowers the content of SA. Our findings indicate that OBPs not only transport odorant molecules [23,25] and sense humidity in the environment [57] but also carry out novel, unrelated functions. Our approach provides a new idea to control BPH with OBPs and insight into the functions of other OBPs.

## 4. Materials and Methods

### 4.1. Plant Materials and Insects

The rice variety Zhonghua11 was used for experiments, and plants were grown as described by Sun et al. [58]. At 50 to 60 days after planting, plants were collected for electrical penetration graph (EPG), JA and SA analyses. The *Nicotiana benthamiana* were grown in a greenhouse at 24 ± 1 °C, 60 ± 10% relative humidity (RH) and a 12 h light/12 h dark cycle and used at 40 days for experiment. BPHs were collected from rice fields in Wuhan, China, and reared on the susceptible TN1 rice cultivar in a net cage in a greenhouse at 28 ± 2 °C, 80 ± 10% relative humidity (RH) and a 12 h light/12 h dark cycle.

### 4.2. PCR and Sequence Analysis

The full-length cDNA of NlugOBP11(GenBank: MW813955) was obtained by PCR from total RNA isolated from salivary glands of adult BPH. Primers (Supplementary Table S1) were designed based on transcriptomic data from adult BPH salivary glands. The PCR product was cloned into the pClone 007 Blunt vector (Tsingke, Beijing, China) and sequenced. The sequence was BLAST-searched against the non-redundant database on the National Center for Biotechnology Information (NCBI) website (http://www.ncbi.nlm.nih.gov/ accessed on 20 March 2021) to search for homologous genes. The SignalIP 4.1 Server (http://www.cbs.dtu.dk/services/SignalP/ accessed on 20 March 2021) was used to predict the presence of signal peptides. Phylogenetic relationships were determined using MEGA 7.0 (https://www.megasoftware.net/ accessed on 20 March 2021) with the neighbor-joining method.

### 4.3. qPCR and Expression Analyses of NlugOBP11

Quantitative PCR (qPCR) was used to investigate the temporal and spatial expression patterns of NlugOBP11 in BPH. Briefly, total RNA was extracted from insects at different developmental stages (first to fifth instars, and newly emerged adult males and females) and tissues (salivary gland, head without salivary gland, thorax, abdomen, leg and wing from BPH adults). Total RNA was isolated using an RNAiso Plus kit (Takara, Kyoto, Japan) and cDNA was synthesized from all samples using a PrimeScript RT Reagent Kit with gDNA Eraser (Takara, Kyoto, Japan) according to the manufacturer’s instructions. qPCR was performed on an CFX96 Real-Time System (Bio-Rad, Hercules, CA, USA) using TaKaRa TB Green Premix Ex Taq II (Takara, Kyoto, Japan) under the following conditions: denaturation for 5 min at 93 °C, followed by 45 cycles at 93 °C for 10 s and 60 °C for 30 s. The primers were designed with Primer-BLAST (https://www.ncbi.nlm.nih.gov/tools/primer-blast accessed on 20 March 2021), and the primer efficiency was tested (Supplementary Table S1). Relative expression levels were calculated using the 2^−ΔΔct^ method [59], and expression levels of the target gene were normalized against the β-actin reference gene.

### 4.4. Expression of NlugOBP11 in Escherichia coli and Antibody Preparation

The complete NlugOBP11 open reading frame (ORF) was cloned into the pET-30a expression vector, and the recombinant plasmid was transformed into *E. coli* BL21 (DE3) for heterologous expression. The target protein was purified by affinity chromatography using nickel-nitrilotriacetic acid (Ni-NTA) resin (General Electric Company, Boston, MA, USA). The purified protein was used to raise rabbit polyclonal antibodies, which were purified by GenScript (Nanjing, Jiangsu, China).

### 4.5. Western Blotting and Immunohistochemical (IHC) Staining

Protein samples for Western blotting analysis were extracted from BPH salivary glands and rice leaf sheaths from plants with or without BPH damage. The method was performed as described by Ji, Ye, Chen, Zeng, Li, Yu, Li and Lou [21]. Briefly, ~300 4th or 5th instar nymphs were released onto rice plants individually and covered with plastic cages (diameter 6 cm, height 10 cm). The outer sheaths were harvested and homogenized in 3 mL of phosphate-buffered saline in liquid nitrogen. The extract was centrifuged at 12,000× *g* for 5 min at 4 °C, and the supernatant collected and concentrated to 200 μL using a YM-3 Microcon centrifugal filter device (EMD Millipore, Billerica, MA, USA). Plants in empty glass cylinders were used as controls, and NlugOBP11 protein served as a positive control. After adding 4× sodium dodecyl sulphate (SDS)-loading buffer, samples were boiled for 10 min and subjected to 18% SDS polyacrylamide gel electrophoresis (PAGE). Proteins were then transferred onto a polyvinylidene fluoride (PVDF) membrane and 5% nonfat milk was used to block nonspecific binding sites. The blot was probed with antibody (1:2000 dilution), and detection was achieved using a goat anti-rabbit IgG horseradish peroxidase (HRP)-conjugated antibody (Jackson ImmunoResearch, West Grove, PA, USA) at a 1:10,000 dilution. Western blots were imaged using a Chemiluminescence Detection Kit (Bio-Rad, Hercules, CA, USA) with a Molecular Imager ChemiDoc XRS System (Bio-Rad, Hercules, CA, USA).

Salivary gland samples used for IHC staining analysis were extracted from adult BPH. Briefly, salivary glands were dissected in phosphate-buffered saline (PBS) and fixed in 4% paraformaldehyde for 30 min. After washing with PBS, samples were blocked with 10% bovine serum albumin (BSA) at room temperature for 20 min. Preimmune serum and antibody were diluted 1:200 at room temperature for 24 h. After three washes with PBS, cy3-labelled secondary goat anti-rabbit IgG (Jackson ImmunoResearch, West Grove, PA, USA) was added, and samples were incubated at room temperature for 12 h. Before measuring the fluorescence, 100 nM DAPI (4′,6-diamidino-2-phenylindole; Sigma-Aldrich, Saint Louis, MO, USA) was used to stain samples. Fluorescence images were observed using a Zeiss LSM 780 confocal microscope (Zeiss, Jena, Germany).

### 4.6. Double-Stranded RNA (dsRNA) Synthesis and RNA Interference (RNAi)

The dsRNA templates were synthesized by PCR with primers containing the T7 promoter sequence. PCR products, dsRNA was synthesized and purified using a MEGAscript T7 High Yield Transcription Kit (Ambion, Austin, TX, USA) according to the manufacturer’s instructions. A 431 bp dsDNA encoding green fluorescent protein (GFP) was synthesized and used as a negative control for RNAi experiments. All primers for qPCR and dsRNA synthesis are listed in Table S1. Third instar nymphs were anesthetized with carbon dioxide for 10–20 s. A 50 ng dsRNA encoding NlugOBP11 or GFP was injected into the BPH mesothorax using a microprocessor-controlled Nanoliter 2010 injector (World Precision Instruments, Sarasota, FL, USA) as described by Liu et al. [60]. After injection, BPHs were reared on 4–5 leaf-stage TN1 rice seedlings. In order to determine the efficiency of gene silencing following dsRNA injection, levels of OBP11 transcripts in the whole body were measured at 2, 4, 6 and 7 days after injection by qPCR.

### 4.7. BPH Bioassays

To investigate the survival rate of BPH after injection, third instar nymphs were reared on TN1 rice plants or artificial diet. Briefly, injected nymphs were reared on 4–5 leaf-stage rice seedlings for 12 h at 28 °C, 60% RH and a 12 h/12 h light/dark cycle, and living nymphs were selected for biological determination. Ten injected nymphs were released into a large glass tube (diameter 4 cm, height 20 cm) containing ~15 rice seedlings as described above, the number of living BPH nymphs was recorded every day for 7 days, and the experiment was repeated four times.

In the artificial diet experiment, 10 injected BPH nymphs were released into a plastic cage (diameter 2 cm, height 10 cm) which was covered with two layers of stretched parafilm membrane and reared on artificial diet as described by Fu et al. [61]. The number of surviving BPH nymphs was recorded every day, artificial diet was replaced daily, and the experiment was repeated four times.

### 4.8. Analysis of BPH Feeding Behavior

At two days after injection, the feeding behavior of surviving nymphs was recorded using a Giga-4 DC EPG instrument (Wageningen Agricultural University, Wageningen, Haarlem, The Netherlands) as described by Sun, Liu, Zhou, Jin, Liu, Zhou, Zhang and Wang [58]. The EPG waveforms of BPH on rice were recorded in a Faraday cage with Style + b software ((Wageningen Agricultural University, Wageningen, Haarlem, The Netherlands) for 9 h, and waveforms for the first 6 h were used for data analysis. All experiments were carried out at 28 ± 2 °C and 60 ± 10% RH. The recorded waveforms were classified into five typical waveforms based on the stylet penetration behavior [38,39]; non-penetration (np), pathway (ph), sieve element salivation (N4-a), ingestion in phloem (N4-b), and xylem sap ingestion (N5). To determine the effect of NlugOBP11 on BPH feeding behavior, the waveform duration and frequency were measured. The experiment was performed at least 7 times.

### 4.9. JA and SA Analysis

Rice plants were damaged with nymphs to induce JA and SA production. Briefly, at 2 days after injection, twenty surviving nymphs were released onto rice plants individually covered with plastic cages (diameter 6 cm, height 10 cm). Plant phytohormones were extracted after 4, 8, 16 and 24 h of nymph damage. Plant phytohormones were quantified by high-pressure liquid chromatography (HPLC) mass spectrometry (MS), with D4-SA and dihydrojasmonic acid added as internal standards during the JA and SA extraction process. Plant phytohormones were extracted as previously described by Pan et al. [62]. For each treatment, four to nine replicates were included.

Chromatography was performed on a ThermoFisher Ultimate 3000 (Thermo Fisher Scientific, Waltham, MA, USA). Separation was achieved on a thermo Accucore C18 column (50 × 4.6 mm, 1.8 µm, Thermo Fisher Scientific, Waltham, MA, USA). A flow rate of 0.4 mL min^−1^ and a column temperature of 40 °C were set for separation of biological samples. Water was the mobile phase A, acetonitrile the mobile phase B, and acetic acid 0.04% *v/v* was used as additive in both eluents. The step gradient was as follows: 98%A and 2%B—2%A and 98%B (linear) during 0–6 min, 2%A and 98%B during 6–8 min, 2%A and 98%B—98%A and 2%B (linear) during 8–8.1 min, 98%A and 2%B during 8.1–10 min. The injection volume was 5 μL, and the samples were held at 4 °C throughout the analysis.

ThermoFisher TSQ Altis triple quadrupole mass spectrometry (Thermo Fisher Scientific, Waltham, MA, USA) equipped with a heated electrospray ion source (HESI) was operated in the negative ionization mode. The HESI parameters were optimized as follows: sheath gas flow rate 40 arb; auxiliary gas flow rate 10 arb; sweep gas flow rate 1 arb; ion transfer tube temp 325 °C; vaporizer temp: 350 °C; spray voltage −3500 eV. Selective reaction monitoring was used to monitor analyte parent ion → product ion: mass-to-charge ratio (*m*/*z*) 136.9 → 92.9 (collision energy (CE), −20 V) for salicylic acid; *m*/*z* 140.9 → 97.0 (CE, −20 V) for D4-salicylic acid; *m*/*z* 209 → 59.0 (CE, −10 V) for JA; *m*/*z* 211 → 59.0 (CE, −10 V) for 9,10-D2-9,10-dihydrojasmonic acid. The peak area of the phytohormones divided by the corresponding internal standard peak area was used to obtain the relative content of phytohormones.

### 4.10. NlugOBP11 Overexpression in Rice Protoplasts and Nicotiana Benthamiana Leaves

The NlugOBP11 sequence without a signal peptide and stop codon was amplified by PCR, and the product was cloned into the pCNG vector (containing a 2 × 35 s promoter and an enhanced GFP tag) and sequenced. The recombinant plasmid was transformed into *E. coli* DH5α and *Agrobacterium tumefaciens* EHA 105. Using an Endotoxin-free Plasmid Midi Kit D6915 (Omega, Norcross, GA, USA), plasmid was extracted and transfected into rice protoplasts as described by Zhang et al. [63]. Total RNA was extracted from rice protoplasts using an RNAiso Plus kit (Takara, Kyoto, Japan), and the expression of genes related to JA and SA pathways were measured at 8, 12 and 24 h by qPCR (primers used to amplify genes are listed in Table S1). Empty vector was transfected into rice protoplasts as a negative control. The experiment was performed three times.

The recombinant *A*. *tumefaciens* strains were cultured in Luria-Bertani medium supplemented with 50 mg mL^−1^ kanamycin and 50 mg mL^−1^ rifampicin. After about 48 h, the strains were collected and resuspended in infiltration medium (10 mM MES, pH 5.6, 10 mM MgCl_2_, and 100 mM acetosyringone), to an OD600 of 1.0. The resuspended recombinant strains were incubated for no less than 3 h at room temperature and infiltrated into the leaves of 4- to 6-week-old *N. benthamiana* plants through a nick created using a needleless syringe. Total RNA was extracted from infiltrated *N. benthamiana* leaves, and expression of genes related to JA and SA synthesis were measured at 24 and 48 h by qPCR (primers used to amplify genes are listed in Table S1). The protein expression levels of NlugOBP11 and GFP in *N. benthamiana* leaves were detected by Western blot as described above and the leaf cells were observed by fluorescence microscope (OLYMPUS IX71, Tokyo, Japan) at 48 h. Empty vector was transfected into *N. benthamiana* leaves as a negative control. The experiment was performed three times.

### 4.11. Statistical Analyses

One-way analysis of variance (ANOVA) was used to analyze the expression levels of genes in different tissues and developmental stages. The differences between the means were determined by Tukey’s HSD test. Student’s t-tests were used to analyze differences in BPH mortality rate, EPG data, JA and SA levels, and expression levels of genes related to JA and SA between treatments. Differences were considered significant at *p* < 0.05, and all data were analyzed using SAS version 9.0 (SAS Institute; http://www.sas.com/ accessed on 20 March 2021).

## Data Availability

The data presented in this study are available on request from the corresponding author.

## Supplementary Materials

The following are available online at https://www.mdpi.com/article/10.3390/ijms22094988/s1, Figure S1. Protein alignment of OBP from insects; Figure S2. Phylogenetic tree for amino acid sequences of OBP11 and reported insect endogenous OBPs; Table S1. Primers used for PCR and qPCR.
