# Supplementary material for: A Salivary Odorant-Binding Protein Mediates Nilaparvata lugens Feeding and Host Plant Phytohormone Suppression
Source: Int J Mol Sci. 2021 May 8;22(9):4988. doi: 10.3390/ijms22094988 (PMC8125829; doi:10.3390/ijms22094988)
Supplement: Supplementary file 1 [file ijms-22-04988-s001.zip › ijms-1181442-supplementary.pdf]

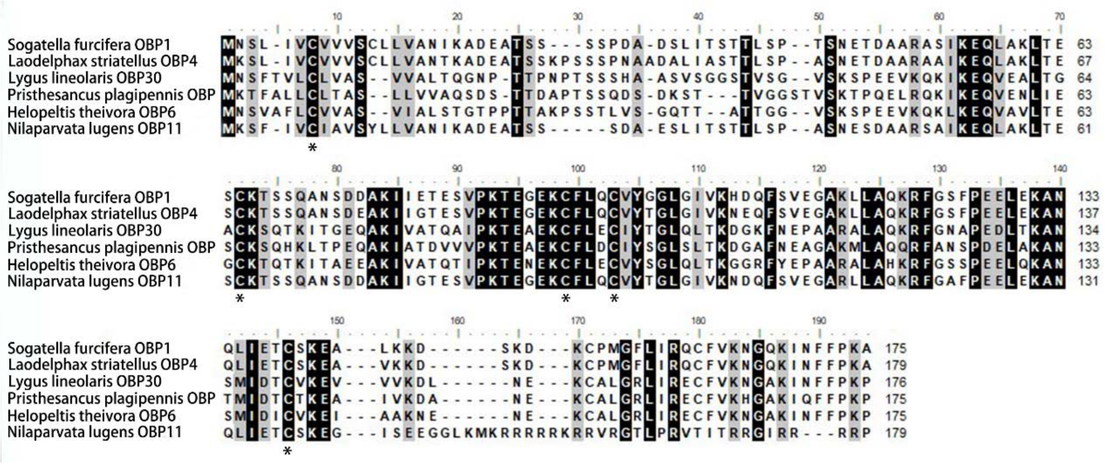

Figure S1. Protein alignment of OBP from insects. *Sogatella furcifera* OBP1(AHB59655), *Laodelphax striatellus* OBP4 (AGZ04923), *Lygus lineolaris* OBP30 (AHF71061), *Pristhesancus plagipennis* OBP (ATU82918) , *Helopeltis theivora* OBP6 (QHI06952). The colors of the aligned sequences showed the degree of amino acid similarity, including identical (black), highly conserved (dark gray) and conserved residues (gray). The position of cysteine residue marked by asterisk.

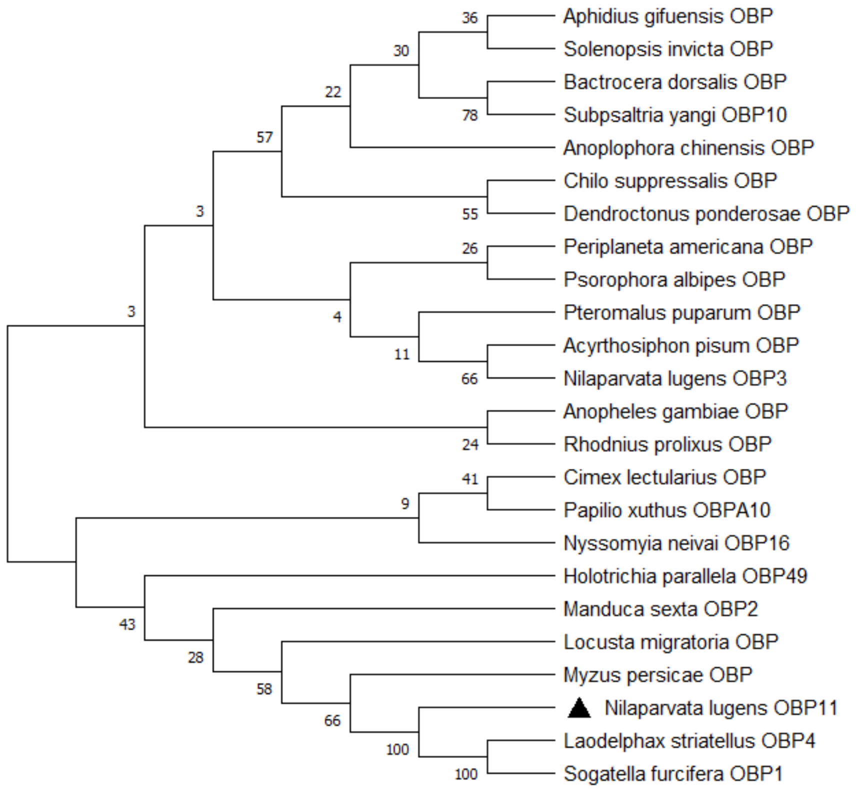

Figure S2. Phylogenetic tree for amino acid sequences of OBP11 and reported insect endogenous OBPs. GenBank accession numbers of the sequences: *Aphidius gifuensis* OBP (AZQ25006), *Solenopsis invicta* OBP (ACI30693), *Bactrocera dorsalis* OBP (ACB56577), *Subpsaltria yangi* OBP 10 (AXY87869), *Anoplophora chinensis* OBP (AUF72991), *Chilo suppressalis* OBP (AGM38613), *Dendroctonus ponderosae* OBP (AFI45063), *Periplaneta americana* OBP (ACI30687), *Psorophora albipes* OBP (JAA94281), *Pteromalus puparum* OBP (AJW76495), *Acyrthosiphon pisum* OBP (NP_001353810), *Nilaparvata lugens* OBP3 (ACI30681), *Anopheles gambiae* OBP55 (AAQ16287), *Rhodnius prolixus* OBP (JAA76272), *Cimex lectularius* OBP (ACY69950), *Papilio xuthus* OBP A10 (NP_001299656), *Nyssomyia neivai* OBP16 (JAV08337),

Holotrichia parallela OBP49 (AKI84360), *Manduca sexta* OBP2 (AAG50015), *Locusta migratoria* OBP (ACI30696), *Myzus persicae* OBP (ACI30682), *Laodelphax striatellus* OBP4 (AGZ04923), *Sogatella furcifera* OBP1(AHB59655).

Supplemental Table S1. Primers used for PCR and qPCR.

Gene	Description	Forward primer	Reverse primer	Primer efficiency
NlugOBP11	qPCR	TGATCACTTCGACGACCCTG	AGAATTGGCTTGGCTGGAGG	0.94
β-actin	qPCR	ACTCCGGTGATGGTGTCTCT	GTCGGTCAAGTCACGACCA	0.98
NlugOBP11	dsRNA synthesis	GGATCCTAATACGACTCACTATAGG GGATCAGTGAAGAAGGAGGAT	GGATCCTAATACGACTCACTATAGGCCGTCTCCTTCTTATTCCTCG	-
GFP	dsRNA synthesis	GGATCCTAATACGACTCACTATAG GCCACCTACGGCAAGCTGACCCTG	GGATCCTAATACGACTCACTATAGGGTCGGCGAGCTGCACGCTGCC	-
NlugOBP11	Protein expression	GGAATTCATGGACGAGGCAACATCTTC	CGCTCGAGTCACGGCCGTCTCCTT	-
GAPDH	qPCR	AAGCCAGCATCCTATGATCAGATT	CGTAACCCAGAATACCCTTGAGTTT	0.98
EDS1	qPCR	CATTCCAAGAACGAGGACACTG	CAAGACTCAAGGCTAGAACCGA	0.96
PAD4	qPCR	CCAACATGTACCGCATCAAG	GGTTGTTTCGGTGGTAGTGG	0.98
PAL	qPCR	GCACATCTTGGAGGGAAGCT	GCGCGGATAACCTCAATTTG	0.96
ICS1	qPCR	TATGGTGCTATCCGCTTCGAT	CGAGAACCGAGCTCTCTTCAA	0.98
LOX	qPCR	GCATCCCCAACAGCACATC	AATAAAGATTTGGGAGTGACATA	0.95
AOS2	qPCR	CTCGTCGGAAGGCTGTTGCT	ACGATTGACGGCGGAGGTT	0.97
L25	qPCR	CCCCTCACCACAGAGTCTGC	AAGGGTGTTGTTGTCCTCAATCTT	0.98
NbLOX	qPCR	AAAACCTATGCCTCAAGAAC	ACTGCTGCATAGGCTTTGG	0.94
NbPR1	qPCR	TGAGATGTGGGTCGATGAGA	CGAGTTACGCCAAACCACTT	0.96
NbPR3	qPCR	TGGGTTATTGCTGGCTTAG	GGGTCATCCAAAACCAGAGA	0.96
NbPR4	qPCR	GGCCAAGATTCCTGTGGTAGAT	CACTGTTGTTTGAGTTCCTGTTCCT	0.97
NbICS	qPCR	GCAAAGCACTGCACTCTACG	GCCTTGGCACCCATTCATTG	0.97
